# To Stop Nose-Bleeding

**Published:** 1898-04

**Authors:** 


THE injection of a glass syringeful of lemon juice into the nose after it has been cleansed of clots, will stop bleeding after everything else has failed.—*Med. Summary.*

